# Management of sleep disorders in autism spectrum disorder with co-occurring attention-deficit hyperactivity disorder: update for clinicians

**DOI:** 10.1192/bjo.2023.589

**Published:** 2023-12-13

**Authors:** Theodore Petti, Mayank Gupta, Yuli Fradkin, Nihit Gupta

**Affiliations:** Rutgers University-Robert Wood Johnson Medical School, Piscataway, New Jersey, USA; Southwood Psychiatric Hospital, Pittsburgh, Pennsylvania, USA; Dayton Children's Hospital, Ohio, USA

**Keywords:** Attention-deficit hyperactivity disorder, autism spectrum disorder, sleep–wake disorder, assessment, treatment

## Abstract

**Aims:**

To update and examine available literature germane to the recognition, assessment and treatment of comorbid autism spectrum disorder (ASD), attention-deficit hyperactivity disorder (ADHD) and sleep disruption, with a predominant focus on children, adolescents and emerging adults.

**Background:**

Considerable overlaps exist among ASD, ADHD and sleep disruption. Literature and guidance for clinicians, administrators, policy makers and families have been limited, as such deliberations were rarely considered until 2013.

**Method:**

This narrative review of the literature addressing sleep disruption issues among those with ASD, ADHD and comorbid ASD and ADHD involved searching multiple databases and use of reverse citations up to the end of September 2022. Emphasis is placed on secondary sources and relevant data for clinical practice.

**Results:**

Complex clinical presentations of ASD/ADHD/sleep disruption are frequently encountered in clinical practice. Prior to 2013, prevalence, clinical presentation, pathophysiology, prognosis, other sleep-related factors and interventions were determined separately for each disorder, often with overlapping objective and subjective methods employed in the process. High percentages of ADHD and ASD patients have both disorders and sleep disruption. Here, the extant literature is integrated to provide a multidimensional understanding of the relevant issues and insights, allowing enhanced awareness and better care of this complex clinical population. Database limitations are considered.

**Conclusions:**

Assessment of ASD symptomatology in youth with ADHD, and the reverse, in cases with disrupted sleep is critical to address the special challenges for case formulation and treatment. Evidence-based approaches to treatment planning and multi-treatment modalities should consider combining psychosocial and biological interventions to address the complexities of each case.

Before modification in the DSM-5, autism spectrum disorder (ASD) and attention-deficit hyperactivity disorder (ADHD) were not allowed to be mutually exclusive diagnoses.^1^ They are among the most prevalent neurodevelopmental disorders, and a growing body of research and clinical experience demonstrates the considerable overlap between them.^2^ Recent publications underscore relationships between these neurodevelopmental disorders and sleep disruption.^3,4^ Most ASD and ADHD empirical research before and shortly after 2013 highlighting clinical, behavioural and cognitive symptomatology conceptualised these disorders as separate developmental trajectories and failed to consider their comorbidity.^5^ This included the 2005 landmark Research Units on Pediatric Psychopharmacology Autism Network trial, which used the teacher-rated Aberrant Behavior Checklist hyperactivity subscale as the primary outcome measure in children with ASD, noting that a large proportion did meet ADHD criteria without the restriction.^6^ Prior to 2013, ASD and ADHD studies were unlikely to consider the comorbidity of the two disorders.^7^ However, past and current research documents substantial relationships between sleep disruption and autism/ADHD symptoms, with connections across the lifespan.^8,9^

Clinically differentiating between ASD and ADHD is complex, owing partly to the entanglement of their symptom descriptions and item overlap,^9–11^ biological risks and neuro-biomarkers, e.g. white matter track deviations.^12,13^ Substantially more dimensionally measured ASD traits are found in youth with ASD compared with those who do not have ASD.^10^ Comorbid ADHD has been reported in at least 50–70% of ASD-diagnosed youth, whereas a recent meta-analysis suggests that 15–25% of those with ADHD meet the criteria for ASD; the numbers are lower but sufficiently significant to warrant clinical attention.^14,15^

Clinically elevated levels of ASD symptoms are present in up to a third of children with a primary diagnosis of ADHD.^16^ Studies of comorbid ASD/ADHD/sleep disruption have not received adequate clinical or research attention to date, even though these disorders are highly comorbid.^17^ Young people with ASD/ADHD experience more daily difficulties compared with those having only one disorder, including lower quality of life and poorer adaptive and cognitive functioning compared with children with only ASD;^15^ they are also less responsive to standard treatments and have greater treatment needs than those with only one disorder.^18^ Other comorbidities of newly diagnosed adults with ADHD, ASD and ASD/ADHD are comparable, except for increased substance use disorders in ADHD.^19^

Sleep disruption most commonly presents as insomnia, e.g. trouble falling or staying asleep, and circadian rhythm dysfunction, i.e. misaligned timing between the external environment and circadian rhythms.^20^ Sleep disruption may precede early childhood diagnosis of ASD.^21^ Sleep disruptions are more commonly present in both ASD and ADHD than in the general population, including specific sleep disorders, e.g. sleep onset insomnia (SOI), obstructive sleep apnoea (OSA), sleep-disordered breathing, restless leg syndrome (RLS) and periodic limb movement disorder (PLMD);^22^ these are likewise described for ADHD.^23^ These sleep-related disturbances continue into adulthood in individuals with ASD/ADHD and represent an important clinical issue to be addressed.^4^ Regrettably, sleep disruptions are infrequently reported, and their care is inconsistently documented in patients with ASD/ADHD/sleep disruption.^24^

Co-occurrence of ASD and ADHD results in greater functional impairments than either individual disorder,^10^ and this appears to be true for sleep disruption in those with ASD/ADHD.^4,25,26^ Moreover, autistic traits in children with ADHD at initial assessment prognosticate a greater burden of psychopathology emerging earlier in life and a compromised course into adult life in multiple domains of functioning, i.e. poorer interpersonal, neurocognitive and educational outcomes.^27^ Our previous paper^22^ reviewed ASD/sleep disruption. This paper reviews empirical research concerning sleep disruptions co-occurring in children and youth with ASD/ADHD.

## Method

We have deviated from the requirement that the manuscript reports a systematic review or meta-analysis of studies following MOOSE guidelines, and it was not prospectively registered via PROSPERO. Instead, this narrative review of sleep disruption among individuals with ASD and comorbid ADHD involved searching multiple databases (PubMed, PsycINFO, Cochrane Library, Google and Google Scholar) and employing reverse citations through to September 2022. Keywords included ‘Autism*’, ‘ASD’, ‘ADHD’, ‘ADD’, ‘Attention Deficit/Hyperactivity Disorder’, ‘Sleep Disturbances’ and ‘Insomnia’. Articles in English from around the world were analysed. Inclusion criteria included articles specifically focusing on sleep difficulties and related issues in individuals with ASD, ADHD and their comorbidity. We also searched both manually and at PubMed Central to find relevant data. Emphasis was given to citing references providing summaries of earlier work, i.e. reviews and meta-analyses. Individual studies are cited when needed or to elaborate upon critical points.

## Results

### Epidemiology, subtypes and clinical manifestations

About 50–83% of children and adolescents aged 2–18 years with ASD are documented to have sleep disruption,^20^ whereas 50–85% with ADHD have been diagnosed with comorbid sleep disruption,^3,4^ particularly those with sensory difficulties.^28^ Pre-2013, pathophysiology, other sleep-related factors and interventions were studied independently, with the objective and subjective methods used sometimes overlapping. SOI has been the most common sleep disruption found in ASD and ADHD studies employing subjective measures. A large body of research documents the frequent occurrence of sleep disruption in ASD^21,28^ and ASD/ADHD.^3,4^ Meta-analysis and other reviews indicate similar sleep-impaired profiles for both disorders compared with healthy controls, with higher sleep onset latency (SOL), greater numbers of awakenings during sleep, poorer sleep efficiency and lower self-perceived sleep quality.^3,4,29^

Sleep disruptions associated with or caused by sensory modulating difficulties or atypical sensory–perceptual disturbances (ASPs) noted in ASD^22^ are also present in children and adolescents with ADHD. In a study of ASP profiles of children aged 8–11 years, comprising 25 children with ADHD, 38 typically developing children (TDC) as controls and 13 children with ADHD and typical sensory profiles, sleep disruption was demonstrated in 86.4% of children with ADHD/ASPs, 30.8% of children with ADHD and typical sensory profiles, and 16.7% of controls. Thus, children with ADHD and typical sensory profiles were indistinguishable from controls, but those with ADHD/ASP had a significantly increased odds ratio for sleep difficulties compared with controls (odds ratio = 32.4; 95% CI 4.0–260.1, *P* = 0.001).^28^

Sleep disorders occurring with high frequency in both ASD and ADHD include bedtime resistance, difficulty falling and staying asleep, irregular sleep–wake cycles, restless sleep and parasomnias.^4^ Discussions concerning ASD/sleep disruption and consequent effects have been more fully explored previously.^22^ For youth with ADHD, SOL is the most prevalent sleep disruption, as determined by objectively measured polysomnography (PSG). Inconsistency of PSG across various sleep parameters in ADHD is explained by changes in rapid eye movement (REM) across developmental stages owing to maturation and night-to-night variability.^4^ A high apnoea/hypopnea index, indicative of sleep apnoea,^29^ and nocturnal motor activity, i.e. periodic limb movements in sleep (PLMS), are the most consistent associations found in children with ADHD. Total sleep time (TST) as measured by PSG and actigraphy is notable for significant discrepancies in comparison with that of TDC, possibly owing to intermittent awakenings.^4^

Two of five actigraphy parameters (SOL and sleep efficiency) and seven of nine subjective symptoms (SOL, higher number of night awakenings, daytime sleepiness, psychosomatic symptoms during sleep onset, sleep quality, general sleep problems and sleep efficiency) were found to be significantly increased in a systematic review and meta-analysis of 13 studies comparing adults with and without ADHD. No significant differences were found in restorative sleep value, sleep duration or PSG parameters between the two groups.^30^ These results indicate substantial continuity of sleep disruption throughout the lives of ADHD individuals.

Greater sleep periodic limb movements are specific to ADHD in adults, whereas a higher proportion of earliest, brief (N1) sleep was found in participants with ASD.^3^ Adults with ADHD report more psychosomatic symptoms during sleep onset, daytime sleepiness, insomnia and a lower perception of being rested at wake-up.^3^ This is similar to the findings of meta-analytic studies of children diagnosed with ADHD with exclusion criteria of psychopharmacologic treatment and presence of anxiety and depression: significantly higher findings for bedtime resistance, night awakenings, difficulties with morning awakenings, more sleep onset difficulties, daytime sleepiness and sleep-disordered breathing compared with TDC controls.^29^

### Intrinsic effects of ADHD and ASD on sleep

Sleep disruption appears to have a greater impact on emotional, behavioural and cognitive difficulties in ASD/ADHD than in either disorder alone. Studies using objective sleep measures, i.e. PSG, in children with diagnosed ASD/ADHD are scarce. However, studies using subjective parental reports indicate sleep problems as more pronounced in comorbid ADHD and ASD.^31,32^ It has been speculated that the bidirectional nature of the effects of ASD and ADHD on sleep disruption is an expression of the intrinsic deficits of these disorders. However, given the similarities, e.g. sleep problems, in each disorder and their increased severity in combined ADHD and ASD, a common underlying pathophysiology may be operating to interrupt the transition from the state of stimulus-seeking alertness leading to SOL and the passive sleep state.

The expanding literature associating sleep problems in children with ASD and symptoms of ADHD post-DSM-5 has suggested bidirectional relationships between inattentiveness and poor sleep quality in ASD.^22,33^ Inattention symptoms may predict an irregular sleep–wake cycle; conversely, sleep disruption may exacerbate daytime inattentiveness.^22,33^ Significantly higher inattention parent ratings predicted higher scores on the Children's Sleep Habit Questionnaire (CSHQ), indicating moderate-to-severe sleep problems, compared with a control group, whereas elevated hyperactivity ratings did not.^33^ Moreover, the extent of the influence of ASD on sleep disruption in ASD/ADHD has been questioned since a large Australian study^34^ failed to find youth with ADHD/ASD to have elevated sleep disruption relative to the ADHD group. These findings indicate that comorbid ASD in children with ADHD may not exacerbate behavioural sleep based on the molecular endophenotype data considered below.^35^ However, more studies are needed to support these counterintuitive findings.

Lifespan psychopathology and sleep problems continue to be strongly related to both ASD and ADHD. Mixed results characterise the interplay between sleep alterations and psychiatric comorbidities in patients with ADHD and ASD.^22,36^ Across all ages, hyperactivity especially relates to reduced TST, increased wake-after-sleep onset and parasomnias. Combined hyperactivity and inattention symptoms are correlated with generally poor sleep, including daytime sleepiness, wake-after-sleep onset and reduced TST. Significant sleep difficulties affect attention and daytime functioning and may result in behavioural changes consistent with ADHD diagnosis.^8^

### Other associated conditions

Sleep-related movement disorders, RLS and PLMD are often associated with daytime sleepiness, poor sleep efficiency and sleep maintenance insomnia; they are less common in the general population than in those with ASD and/or ADHD. Several explanations have been offered for sleep disruption in comorbid ASD/ADHD. One is related to low serum ferritin contributing to RLS and/or PLMD as an indirect result of nutritional deficiencies, secondary to the adverse effect of appetite suppression of stimulant medications. As youths with ASD and ADHD often have communication deficits, clinically diagnosing PLMD in these children based on self-reported symptoms is challenging.^4,37^

Sleep-related breathing disorders include sleep-disordered breathing and OSA. Studies have documented their presence to significantly worsen ADHD and ASD symptoms. Sleep disruptions may exacerbate daytime inattentiveness, or, conversely, inattention symptoms may predict an irregular sleep–wake cycle. Risk factors include obesity, low muscle tone and motor delays, which are all common in both ADHD and ASD but are more prevalent in combined ADHD/ASD. Obesity could be secondary to medications, e.g. atypical antipsychotics or mood stabilisers for the treatment of aggression and irritability in youth with ASD and ADHD, and to abnormal or restrictive eating habits.^4^

Parasomnias reported in both ASD and ADHD are related to changes in non-REM (NREM) sleep, increased fragmentation of sleep and scarcity of REM sleep. They include ‘confusional arousals’, sleepwalking as a form of increased motor activity, night terrors, wake screaming and enuresis. Rare cases of REM behaviour disorders attributed to medications have been reported in both disorders. The cyclic alternating pattern, a recurring endogenous rhythm in NREM sleep and often used as a measure of NREM stability, is significantly lower in both disorders. The risk of parasomnias is associated with anxiety and other comorbidities, and with medications used in their treatment.^4^

ADHD individuals are more susceptible to sleep disruption caused by stress, as evidenced by inter-pandemic data comparing youth with ADHD or ASD with TDC. Those with ADHD demonstrated greater instability of sleep schedule and later bedtimes that may represent distinctive markers of the ADHD condition.^38^

Although narcolepsy is not a formal sleep disruption diagnosis, it is most often found to be comorbid with sleep disruption that sometimes masks its diagnosis. This is particularly true for ADHD, representing a specific phenotype,^39^ with over 30% of individuals with narcolepsy so diagnosed;^37^ it is less so for ASD, and by extension for youth and adults with ASD/ADHD/sleep disruption. Delayed diagnosis may be attributed to markedly different broad, varying symptom presentation between adults and children, and to masking by other related sleep disruptions with overlapping symptoms and treatment.^40,41^

Gastrointestinal disorders represent another set of comorbidities that may affect sleep in both disorders. According to parent reports, 85% of 118 youth with ASD/ADHD in a recent study experienced at least one gastrointestinal symptom within the previous 3 months, without a significant difference in total gastrointestinal symptoms between those with ASD only and those with ASD with ADHD symptoms. In the same study, 91.5% of youth presenting with CSHQ reported sleep disruption; daytime sleepiness was the most frequently reported sleep problem across age groups. Those with only ASD had fewer sleep problems than those with ASD/ADHD.^17^

The impact on sleep of the presence of intellectual disability in ASD and ADHD is uncertain; some studies suggest that children with ASD with more severe levels of intellectual disabilities are more likely to have increased sleep disruption than healthy individuals, but other studies found no effect.^33,42,43^

Directly and indirectly, sleep disruptions and other symptoms of ASD/ADHD are associated with poorer family function and subsequent increased parental stress and poorer mental health; moreover, there are bidirectional effects of parental stress and sleep problems in children, resulting in a less than positive environment for emotional and other development.^44^ Barriers to effectively treating insomnia in paediatric ASD/ADHD and other neurodevelopmental disorders include a dysfunctional home environment combined with associated exhaustion and limited capacity to implement treatment.^45^ Maternal autism traits, lower paternal education and family income may affect sleep disruption in neurodevelopmental disorders.^43^

### Neurobiological basis

#### Co-occurring and interacting variables

Understanding the cause of these sleep disruptions to improve treatment outcomes remains a clinical priority.^20^ Lifespan psychopathology and sleep problems are strongly related to ASD and ADHD. Mixed results characterise the interplay between sleep alterations and psychiatric comorbidities in patients with ADHD, ASD and other psychiatric disorders, particularly as related to circadian rhythm and related aberrations.^46^ Significant sleep problems interrelated with both behaviour and psychopathology have been demonstrated with genetic underpinnings.^47^ Unlike the ASD/sleep disruption literature, in which significant insight is developing through extensive research,^22^ a relative paucity of research exists for ASD/ADHD/sleep disruption.

**Fig. 1:**
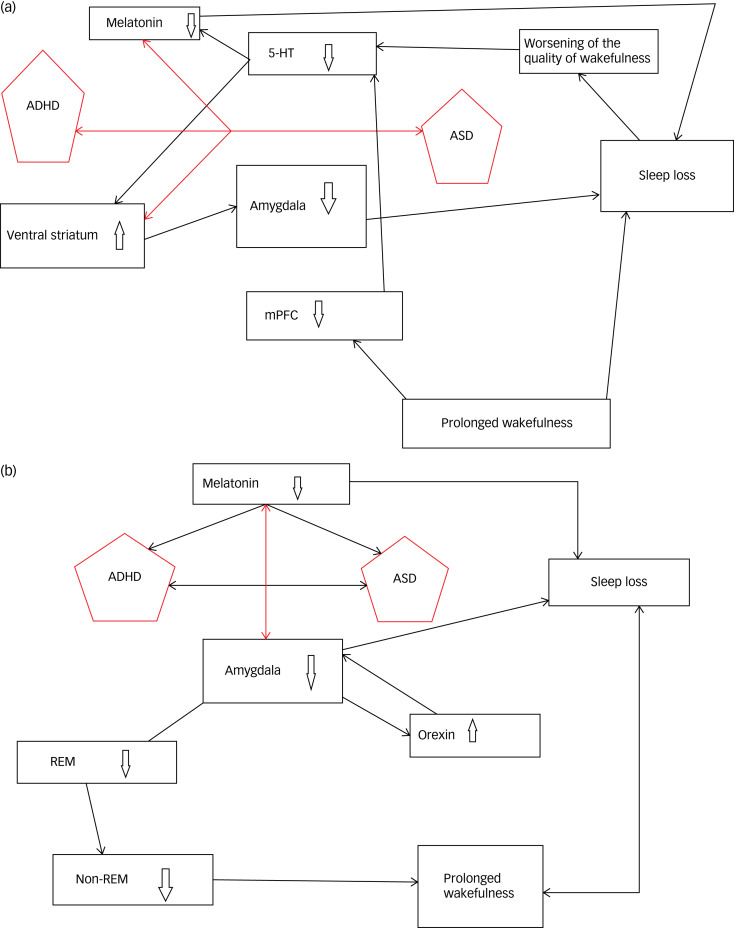
(a) Interactions between effects on sleep of decreases in melatonin caused by the melatonergic system and 5-hydroxytryptamine (5-HT) activity in patients with attention-deficit hyperactivity disorder (ADHD) and autism spectrum disorder (ASD). (b) Interactions between effects on sleep of increased orexin in the melatonergic system and 5-HT activity in patients with ADHD and ASD. mPFC, medial prefrontal cortex; REM, rapid eye movement.

Kohyama^48^ has proposed three neuronal mechanisms involved in insomnia in ASD and ADHD requiring further attention: increased orexinergic system activity; reduced melatonergic system and 5-hydroxytryptamine activity, including dysregulation of the serotonergic signalling system; and reduced REM sleep. The 5-hydroxytryptamine system may influence the melatonergic system, which contributes to the regulation of the sleep–wake cycle and may be influenced by the decreased quality of awake time worsened by sleep loss. Prolonged wakefulness has been shown to reduce medial prefrontal cortex activity and disinhibit functional amygdala activity in ASD. In normal control subjects, total sleep deprivation increased ventricular striatum activity elicited by rewarding stimuli. In ADHD patients, insomnia may be initiated by ventricular striatum activation, which produces amygdala deactivation, causing further stimulation of the orexinergic system, which produces prolonged wakefulness.^48^ The complex association suggests a multi-factorial etiology.^49^ Figures 1(a) and 1(b) depict these interactions.

Patients with ASD and ADHD share common genetic variances through childhood and adolescence. *De novo* mutations, copy number variations (CNVs) and common genetic variations from genome-wide association studies (GWAS) have all been implicated in the genetic aetiology of ASD. Similar rare variants, CNVs and GWAS single nucleotide polymorphisms have been implicated through anomalies of a gene set labelled CLOCK (circadian locomotor output cycles kaput) genes; these anomalies are specifically linked to sleep disorders in ASD/ADHD.^50^ Charrier and associates consider these linkages and the numerous genes involved; however, these are too detailed to discuss in this review.^47^

Circadian rhythm plays a major part in sleep disruption. It is generated by a master central clock in the suprachiasmatic nuclei (SCN), with regulation processed through direct or indirect signals between the SCN and various body structures by peripheral clocks that optimise each organ's function to adapt to environmental changes. Cyclic gene expression and rhythmic physiological processes, controlled by central and peripheral clocks, work independently but require continuous resynchronisation through the brain's master clock. Melatonin, which is inhibited by light exposure and controlled by the SCN master clock, is involved in these synchronisations of peripheral oscillations, synthesis, and release of melatonin by the pineal gland. ASD and ADHD are linked to single nucleotide polymorphisms in core circadian clock genes.^47^

The molecular mechanisms underlying the circadian rhythms include enhancer elements, repressor elements and control loops involving phosphorylation, dephosphorylation, methylation, acetylation reactions and specific protein dimerisation. The molecular circadian system is present in the hypothalamic central clock and the SCN, and in secondary clocks within the brain and peripheral organs. Transcriptional regulation of genes at the DNA level and post-transcriptional regulation, downstream of transcription, are mechanisms implicated in the circadian clock's molecular autoregulatory feedback loop.^47^

#### Genetics, genomics, melatonin and the endocannabinoid system (ECS)

Recent genetics and genomics research documents greater attention to CNVs in ADHD, ASD and other neurodevelopmental disorders.^51,52^ Decreases and increases in gene expression caused by both loss and gain of genetic material represented by CNVs are associated with ASD/ADHD/sleep disruption. ASD's heterogeneous genetic background can be explained by multiple genetic abnormalities, which are different in each individual but converge towards the same biological network. Different pathways for ASD can be roughly grouped into pathways related to CNV alterations associated with cell proliferation, signal transduction, apoptotic signals and brain development shared by different neurodevelopmental disorders comorbid with ASD.^35^

ADHD and other comorbid behavioural symptoms, e.g., sleep disruption, common in ASD, can originate from alteration patterns including abnormalities in the regulation of neural precursor cell proliferation, regulation of smoothened signalling pathways, spinal cord development, stem cell proliferation, neuron fate specification, circadian rhythm regulation, the diencephalon and endocrine system development. These pathways have been grouped into nine CNV clusters.^35^ Table 1 details the relevance of these clusters to ASD/ADHD/sleep disruption and possibly explains the undue influence of ADHD on their impact on sleep behaviours and sleep disruption symptoms that are potentially related to circadian rhythm.^38^

**Table 1:** Relevance of copy number variation (CNV) alterations to autism spectrum disorder (ASD), attention-deficit hyperactivity disorder (ADHD) and sleep disruption (SD) in patients with ASD. Pathways of nine overlapping CNV clusters relevant to ASD/ADHD/SD are shown, emphasising large contributions that may explain the undue influence of the ADHD cluster on sleep behaviours and SD potentially related to circadian rhythm (Briuglia, 2021)

ADHD, behavioural anomalies and circadian rhythm alterations	Regulation of smoothened signalling pathway Regulation of circadian rhythm
Overlapping pathway clusters between ADHD and circadian rhythm alterations	Regulation of smoothened signalling pathway Spinal cord development Neuron fate specification Regulation of circadian rhythm
Overlapping pathway clusters between ADHD and behavioural anomalies	Regulation of neural precursor cell proliferation Regulation of smoothened signalling pathway Regulation of circadian rhythm Neuron fate specification Spinal cord development Regulation of stem cell proliferation
Behavioural anomalies and circadian rhythm alterations	Regulation of smoothened signalling pathway Regulation of circadian rhythm

Youth with ASD and those with ADHD have disorganised behaviours, faulty transmission of entrainment cues and an inability to transition to the passive state of sleep leading to SOL from the state of stimulus-seeking alertness. Circadian rhythm sleep–wake disorders for insomnia have been documented in both ASD and ADHD. Dim light melatonin onset, a primary marker for determining whether a person is synchronised to a 24 h sleep–wake cycle or not, is present in both disorders through abnormal patterns of melatonin secretion, as are abnormalities in CLOCK genes responsible for circadian rhythm maintenance.^4,47,53^

The impact of melatonin on regulation of the sleep–wake cycle is broad in youth with neurodevelopmental disorders, including ASD/ADHD/sleep disruption, and comprises altered circadian rhythms; altered and decreased melatonin production; and abnormal synthesis, concentrations, release patterns and metabolism of melatonin. Altered melatonin production is present in about two-thirds of ASD youth. Further influence results from disturbed signalling of intracellular melatonin receptor type 1A, inflammation of the central and peripheral immune systems, and immune signalling dysregulation.^54,55^ Melatonin pathway dysregulation is likely to be the mechanism driving melatonin variations.^56^

Based on modelling from diffusion magnetic resonance imaging, similar white matter tract deviations have been reported to be shared by ASD and ADHD patients, with inter-individual variability relative to the norm in a degree of deviation.^12^ Likewise, in both disorders, common white matter abnormalities in the splenium of the corpus callosum have been identified, with a wider abnormal pattern noted in ASD.^13^ However, comorbid ASD/ADHD/sleep disruption diagnoses were not considered.

The neuromodulator role of ECS components, which is essential to the regulation of many brain functions, has drawn extensive interest as a potential target for treating ASD, ADHD and other psychiatric disorders. Animal models and human clinical data provide a cornucopia of data relevant to this area of molecular and genetic importance concerning ASD and ADHD. Although extended attention to this subject is impractical here, details are available in the citations that follow. Potentially useful biomarkers relating to alterations in ECS components include expression of cannabinoid receptor genes (CNR1, CNR2), which encode the cannabinoid receptors (CB1R and CB2R).^57–60^ CNS alterations related to ECS in the cerebellum, basal ganglia and hippocampus affect both ASD and ADHD.^57,61,62^

### Interventions

Treatment considerations in approaching and formulating interventions are similar for individual ASD, ADHD and sleep disruption. They should be based on comprehensive multi-modal assessment in individuals manifesting ASD or ADHD symptoms comprising subjective and objective measures.^4,22,37,63–68^ The overall treatment goal is to consider the underlying core deficits contributed by each component and not rely on approaches typical for the comorbid disorder. Rametkkar^4^ reasonably asserts that failure to do this could result in ineffective treatment or possibly worsen sleep issues. Approaches include sensory, behavioural, non-pharmacological and pharmacological interventions; ongoing collaboration with multiple agencies, including public and private educational services; and parent education and support. The lack of controlled studies for ASD/sleep disruption and ADHD/sleep disruption remains problematic.^66^ Parent education, support and training must be considered key components.^37,52,66,69^ Interventions delivered virtually have promise.^37,70^ Table 2 summarises considerations that should guide interventions.

**Table 2:** Summary of recent evidence on autism spectrum disorder (ASD), attention-deficit hyperactivity disorder (ADHD) and sleep disruption (SD) with clinical implications

Author(s)	Study findings	Clinical implications	Interventions
Antshel et al (2016), Ramtekkar (2017), Rong et al (2021)	ASD symptoms are present in one-third of children with ADHD.	Lower quality of life and poorer adaptive functioning in youth with comorbid disorders as compared with those with only one disorder, as well as less responsiveness to standard treatments, and greater treatment needs than those with only one.	Early recognition and assessment of these individuals is critical to treatment planning, particularly when sleep dysfunction is present.
Hollingdale et al (2020)	Co-occurrence of ASD and ADHD results in greater functional impairments.	Compromised course into adult life in multiple domains of functioning, i.e. poorer interpersonal, neurocognitive and educational outcomes.	Long-term follow-up care is needed as impairments continue into adult life.
Ballester et al (2020)	50–83% of those aged 2–18 years with ASD have SD	Higher SOL, greater number of awakenings during sleep, poorer sleep efficiency and lower self-perceived sleep quality.	Screening questions to help identify and stratify specific SD areas allow planning of interventions accordingly.
Ramtekkar (2017), Lugo et al (2020), Mimouni-Bloch et al (2021)	50–85% with ADHD have been diagnosed with comorbid s.d. and particularly in those with sensory difficulties	Higher SOL, a greater number of awakenings during sleep, poorer sleep efficiency, and lower self-perceived sleep quality	Identifying overlapping nature of s.d. in ASD and ADHD is critical for optimal care.
Memouni-Bloch et al (2021), Gupta et al (2023)	SDs are associated with or caused by sensory modulating difficulties or ASPs	ASPs are present in both ASD and ADHD children and adolescents and increase sleep dysfunction.	Screening for ASPs is important; their presence demands consideration in treatment planning.
Ramtekkar (2017)	Bedtime resistance, difficulty falling and staying asleep, irregular sleep–wake cycle, restless sleep and parasomnias are prevalent in both ASD and ADHD.	For ADHD youth, SOI is variably reported as the most common objectively measured PSG. Inconsistency of PSG across various sleep parameters in ADHD has been explained by changes in REM across developmental stages due to maturation and night-to-night variability.	Psychoeducation about SOI and its developmental trajectories may reduce parental anxiety and facilitated implementing evidence-based strategies with behavioural plans to mitigate these issues.
Ramtekkar (2017)	PLMS are the most consistently found associations in children with ADHD.	Total sleep time measured by PSG and actigraphy in comparison with TDC is notable for significant discrepancies, possibly owing to intermittent awakenings.	The clinician recognition of these differences could help in early detection of these cases.
Cortese et al (2009)	High apnoea/hypopnea index is indicative of sleep apnoea.	AHI ≥ 5, mild; AHI ≥ 15–30, moderate; AHI > 30, severe.	OSA is highly comorbid and requires assessment and treatment.
Díaz-Román et al (2018)	Five specific parameters were compared in adults with and without ADHD.	No significant differences were found in restorative sleep value, sleep duration or PSG parameters between the two groups.	There is a substantial continuity of SD through the life of ADHD individuals, and symptoms fluctuate; therefore, ongoing psychoeducation is needed.
Lugo et al (2020)	Greater sleep periodic limb movements are specific to ADHD in adults, whereas a higher proportion of earliest, brief (N1) sleep was found in ASD.	These differences in clinical presentation underscore phenotypic heterogeneity and challenges for treatment.	Clinicians need to be aware of these complex clinical co-occurring conditions, which need comprehensive treatment planning and long-term monitoring.

AHI, apnoea/hypopnea index; ASP, atypical sensory–perceptual disturbance; OSA, obstructive sleep apnoea; PLMS, periodic limb movements in sleep; PSG, polysomnography; REM, rapid eye movement; SOI, sleep onset insomnia; SOL, sleep onset latency; TDC, typically developing children.

Given the delayed acceptance of comorbid ASD/ADHD diagnoses until 2013, significant gaps in the emerging evidence have hindered meeting the clinical needs of these highly complex patient populations. Although it has recently been argued that the existing literature for ASD/ADHD/sleep disruption interventions for sleep difficulties, including behavioural therapies, sleep hygiene and melatonin use, is of low quality,^37^ this update indicates progress in the past few years that softens this conclusion.

Interventions begin best by first recognising the significant prevalence of sleep disorders and other comorbidities^37^ in ASD and ADHD in order to diminish the risk of misattributing symptoms of inattention and hyperactivity to the primary diagnoses. Approaches to distinguish between sleep disruptions in clinical practice, and treatment rationales guiding differing dosages and times of administration, follow.^37,54^ This allows for accurate case formulation of predisposing, precipitating, perpetuating, and positive or preventive factors, and appropriate psychosocial treatment with or without pharmacological interventions,^67^ including collaboration with sleep specialists when needed.^23^ A meta-synthesis of multiple therapeutic interventions concluded that compared with other interventions for ameliorating multiple ASD/sleep disruption domains of sleep problems, melatonin, behavioural interventions and education/parent education appear to be the most effective. However, across all ASD sleep problems, no single intervention was found to be effective.^69^ Recent recommendations offer considerable guidance for identification, assessment, interventions and service needs for children and adults with comorbid ASD/ADHD/sleep disruption and can be summarised as follows.

The high ASD/ADHD co-occurrence rate means both conditions should be considered if one is present; this is particularly important for ASD, as it occurs earlier and clinical improvement over time is less. In adolescents and emerging adults, where symptoms may not have warranted earlier referral, the comprehensive assessment of the person's functioning should span many years. This is particularly relevant for females with ASD, who may be under-identified owing to presenting with inattentive symptoms and minimised disruptive behaviour, higher prevalence of intellectual disability and screening by scales based predominantly on male samples.^52^

**Fig. 2:**
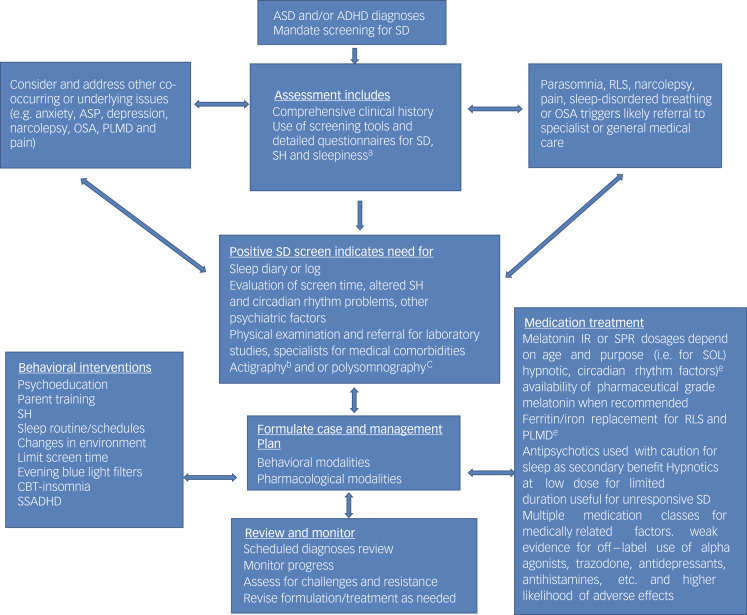
Flowchart describing approach to the complex developmental phenotypes of autism spectrum disorder (ASD), attention-deficit hyperactivity disorder (ADHD) and sleep disruption (SD). ^a^Specific scales are available for SD (Children's Sleep Habit Questionnaire (CSHQ; CSHQ-autism); Pittsburgh Sleep Quality Index (PSQI)), sleep hygiene (SH; Adolescent Sleep Hygiene Scale) and excessive daytime sleepiness (Epworth Sleepiness Scale). ^b^Two weeks movement-based data could add to assessment but does not substitute for clinical evaluation. ^c^Gold standard to detect such complex presentations as narcolepsy, periodic limb movement disorder (PLMD)/ restless leg syndrome (RLS), obstructive sleep apnoea (OSA) and complicated parasomnias, mostly recommended by sleep specialists. ^d^Low-dose melatonin 0.5–1.0 mg as a chronobiotic 3–5 h before bed. Hypnotic dose 3–6 mg 30–60 min before bed. Melatonin slow/prolonged release (SPR): effective for refractory insomnia. ^e^Supplement iron for ferritin <50 ng/mL. ASP, atypical sensory–perceptual disturbance; CBT, cognitive–behavioural therapy; IR, immediate release; SOL, sleep onset latency; SSADHD, Sleeping Sound with ADHD. Modelled on a figure from Shanahan et al. (2021) under terms of the Creative Commons Attribution License.

Several publications offer detailed tables, boxes and figures, providing clinicians with specifics and insights too numerous to present here. They consider common, highly relevant clinical issues previously described.^22,67^ These comprise a range of rating scales and questionnaires, including those free of charge asterisked; guidance for clinical assessment and its reporting, including critical, often overlooked historical and contextual items (e.g. diet, environment); types of sleep disruption (e.g. somnambulism or night terrors, sleep apnoea, RLS, rhythmic movement disorder), with descriptions, and symptoms and signs indicating referral need; treatment planning; social transitions; related mediating and moderating factors affecting outcome; general service needs; pharmacological and non-pharmacological clinical interventions; and practice recommendations for education and/or school interventions.^4,52,66,68^ Figure 2 provides a flow chart showing an overview of the process from screening for sleep disruption to interventions.^37^

Clinicians need to be prepared to use many of the advances that have occurred in the past few years, i.e. advances in basic science, behavioural and pharmacological interventions, and assessment instruments not considered in prior reviews and recommendations, such as the CSHQ, a mainstay of clinical practice and research. A modification, the CSHQ-autism is a shorter version, consisting of 23 items with four factors (down from 45 or 33 item versions with eight factors), with cut-off scores from ages 2 to 17 years and better performance in identifying sleep disruption in individuals with ASD.^71^ It has been successfully employed in studies of objective measures and has acceptable psychometric properties with cut-off scores for the paediatric population.^72^

#### Behavioural interventions

Non-pharmacological and behavioural interventions have long been considered the treatment of choice for children and adolescents diagnosed with ASD/sleep disruption ^22^; the overwhelming consensus is that management of insomnia and circadian rhythm disorders begins with non-pharmacological interventions for ASD/ADHD/sleep disruption, and there are established strategies to address SOL and bedtime resistance.^4,37,66,68,73^

Lifestyle factors to address comprise diet and exercise; there are also strategies to ensure adherence, clearly documented instructions and use of personalised reminders, visual schedules, and incentives for positive sleep hygiene practice, i.e. using the bed only for sleeping, consistent place/bed for sleeping, regular sleep–wake schedule, avoidance of electronic media soon before bedtime, structured transition to sleep, calming bedtime routines, and avoidance of caffeine, naps and exercise immediately before bedtime are highly recommended.^4^ Social Stories™ and other positive strategies are recommended owing to their demonstrated short-term effectiveness in reducing behaviours that challenge bedtime routines and facilitating positive sleep hygiene.^37^

Strategies to improve sleep latency and sleep hygiene in adolescents with ASD/ADHD/sleep disruption include online parent training for setting and reviewing homework tasks, stimulus control strategies for environmental facilitation of sleep and use of other places for wakefulness. Light therapy is suggested for adults with ASD/ADHD with circadian rhythm disorders. Additional stress is placed on growing evidence for face-to-face or online use of cognitive–behavioural therapy for insomnia and on functional analysis and function-based intervention for daytime distress and maladaptive behaviours (e.g. restricted and repetitive behaviours) affecting daily living and sleep routines.^37^

More severe sensory issues and motor deficits^16^ and their impact on sleep into adulthood have been demonstrated in both ADHD ^74^ and ASD^75,76^ populations. Sensory-based interventions based on the determined extent and nature of ASP difficulties should be developed from the case formulation to effectively address sleep disruption. SOL, intra-individual variability in sleep parameters and intermittent awakenings have been reduced through stimulating sensory receptors and transmitting inhibitory signals to the central nervous system; weighted blankets or related means are of questionable value.^77^

A paucity of high-quality evidence demonstrating the effectiveness of non-pharmacological interventions for ASD/ADHD/sleep disruption populations is concerning, with small percentages of ASD children with behavioural insomnia reported to respond to combined behaviour therapy and sleep hygiene.^37,54^ However, significant efficacy has been demonstrated for Sleeping Sound with ADHD (SSADHD), a brief behavioural sleep intervention for individuals with ASD/ADHD.^78^

The SSADHD effect size 3 months post-randomisation for decreased total child sleep problems (ES = −0.7, *P* = 0.02) was significant; it was somewhat less at 6 months post-randomisation (ES = −0.5, *P* = 0.08) but still significant. There was similar improvement in SOL according to the CSHQ at 3 months (ES = −0.9, *P* < 0.001), but this did not significantly persist. Parasomnias (ES = −0.6, *P* = 0.04) showed increased improvement (ES = −0.07, *P* = 0.006) at 6 months, and there were delayed improvements in sleep duration (ES = −0.5, *P* = 0.1; and ES = −0.8, *P* = 0.003) at 3 and 6 months, respectively. Insignificant improvements were noted in sleep anxiety, night awakenings and daytime sleepiness. ESSADHD, which is designed to be embedded into current treatment plans, can be employed by mental health clinicians and paediatricians in everyday practice, rather than the several treatment hours that ASD/ADHD/sleep disruption children generally access.^78^

Sunlight and intense natural light treatment for circadian rhythm disorders, using appropriately timed exposure to light to gradually delay the patient's biological clock, is termed bright light therapy. It borders on behavioural and organic approaches. Phototherapy has been successfully documented in adult ADHD/sleep disruption controlled studies; its use in the paediatric population is limited but promising.^37,46^

### Pharmacological interventions

Pharmacological interventions have been employed in each individual ASD/ADHD/sleep disruption comorbidity but are generally not considered as first-line treatments for sleep disruptions. No medication has been approved by the Food and Drug Administration (FDA) for treatment of paediatric insomnia. Psychopharmacology has a critical role as advances are made in understanding the multifaceted complexity of ASD/ADHD/sleep disruption but must be guided by the differential diagnosis (i.e. coexisting medical and/or mental illnesses) and formulation based on predisposing, precipitating and perpetuating factors (e.g. iatrogenic sleep disruption, poor sleep hygiene, pharmacogenetics).^52,79^ Insomnia-risk psychotropic drugs, i.e. those unmasking subclinical REM sleep dysfunction behaviour disorder and those with somnolence as an adverse effect must be considered, as should dosing strategies.^37^

About a third of ASD/ADHD/sleep disruption patients receive pharmacological treatment for comorbid symptoms. In one study, between 30 and 50% of ASD patients treated with medication experienced long-lasting, severe side-effects and/or did not respond adequately; however, melatonin for sleep disruption was not considered in this study or its literature review.^79^ Melatonin is recommended in the most recent practice guidelines,^54^ specifically if the response to behavioural interventions is inadequate.^64^ Moreover, it has been approved by a credible medication-regulating agency outside the USA.^21^

Melatonin is clearly the choice for circadian rhythm sleep–wake disorders and insomnia, as it improves sleep latency, sleep efficiency and total sleep duration. Early studies of immediate-release melatonin for a range of paediatric neurological and developmental disorders with severe sleep disruptions demonstrated efficacy and safety. A 12-week randomised, double-blind placebo-controlled trial documented increased TST and reduced SOL using sleep diaries and actigraphy, with associated improvements in child behaviour and family functioning outcomes, and earlier wake times compared with placebo. No adverse effects or differences between the groups were noted.^54^

A large multicentre collaborative phase III study with adequate sleep hygiene interventions demonstrated highly significant improvement in SOL (as documented using an electronic sleep diary), irritability, inappropriate speech, hyperactivity and stereotypical behaviour. No treatment-emergent adverse effects occurred.^80^

The finding that melatonin significantly shortens SOL has been duplicated in a large, randomised placebo-controlled trial. Fixed doses of immediate-release melatonin (1 mg and 4 mg) were compared with a placebo in children with ASD (about 40% with ADHD) under adequate sleep hygiene interventions following 14 days on placebo.^81^ Limitations of immediate-release melatonin have been mitigated by use of a paediatric-appropriately dosed prolonged-release melatonin formulation. In paediatric ASD/sleep disruption populations, the significant improvement in total sleep duration and continuity achieved with melatonin compared with placebo was unaffected by stimulant use or comorbid ADHD.^22,37,52^ Moreover, its availability as a 3-mm-diameter prolonged-release melatonin minitablet meant it had remarkably high acceptability by a population who usually experience significant difficulties in swallowing.^82^

Melatonin has demonstrated good tolerability and superior efficacy in ASD/sleep disruption, and a newer extended-release formulation is deemed particularly effective, safe and acceptable. Long-term, nightly (up to 104 weeks) use of 2 mg, 5 mg or 10 mg extended-release melatonin in 80 youth aged 2–17.5 years (96% with ASD, completers of a double-blind trial) was also assessed with respect to withdrawal effects during a 2 week placebo period. No detrimental effects on pubertal development or growth, safety issues or withdrawal effects were found during melatonin use or its discontinuation.^22,83^

Likewise, melatonin has had considerable research support for use in ADHD and ASD/ADHD, although caution concerning adverse effects is recommended.^4^ Melatonin influences both chronobiotic (action levels within circadian pacemaker, e.g. retina, hypothalamic tracts, SCN, feedback and output systems) and hypnotic properties affecting circadian rhythm sleep disorders. A recent study involving sleep disruption precipitated by methylphenidate (MPH), has demonstrated melatonin to be effective and safe regardless of comorbidities, age or gender.^84^ Similar findings were reported in a recent review of ASD/ADHD/sleep disruption.^85^

Concerns relate to melatonin product variability in the USA, where it is considered a dietary supplement and not an FDA-regulated drug, as well as accidental ingestion. Ultraperformance liquid chromatography with electrochemical detection for quantification of melatonin and serotonin conducted on 31 melatonin supplement products showed that from −83% to +478% of labelled melatonin content was not correlated with product type or manufacturer. Lot-to-lot, products varied by as much as 465%. More than 70% of supplements failed to meet labelled content within a 10% margin, and 26% were found to contain serotonin at levels of 1 to 75 μg.^86^ Thus, when the efficacy of melatonin is not achieved or wanes, switching brands or brand lots is recommended. Pharmaceutical-grade melatonin is recommended when available.^65,68^

Regrettably, melatonin was the most frequently ingested substance in 2020 by youth aged ≤19 years reported to national poison control centres. It accounted for 4.9% of ingestions during 2021, up substantially since 2012; the majority of cases were unintentional, and most were asymptomatic, but 1.6% had serious outcomes including death in two between 2012 and 2021.^87^

Immediate-release melatonin is recommended for children with difficulty in initiating sleep onset, usually at a dose of 1–3 mg, administered 30 min (range in studies from 20 to 60 min) before bedtime if it is to act as a sleep inducer. The same dose is recommended for sustained-release melatonin for children with difficulty maintaining their sleep. Melatonin is given 3–4 h before sleep if it is used as a chronobiotic.^85^ Dosages remain uncertain.^88^ Consensus-determined doses have been 1 to 2 mg for ages 3–5 years, 2 to 3 mg for 6–12 years and up to 5 mg for adolescents and adults.^89^

Stimulants remain the first-line medication for the treatment of ADHD symptoms. Case formulation should determine positive and negative benefits in balancing their documented insomnia induction with ADHD symptom control, thus facilitating improved functioning to promote sleep; sleep treatment with stimulants, guanfacine and atomoxetine may improve sleep disruption.^37^ Recently, positive or neutral sleep disruption outcomes have included a randomised fixed-dose, double-blind, 4-week placebo-controlled trial of a multilayer, extended-release MPH formulation with an open-label 6-month follow-up. A marginally higher proportion of medicated patients compared with the placebo group went from poor to good sleeper status on the Pittsburgh Sleep Quality Index. The study results showed the expected positive response with respect to ADHD symptoms, and the treatment was not a predictor of poor sleep at double-blind study termination according to logistic regression analysis.^90,91^ Similar responses have been found in adults with ADHD^92^ and more recently in both children and adults.^93^

More recent studies have reported a linear dose–response relationship with MPH for youths with higher-functioning ASD; however, experts suggest using lower doses of MPH for youths with lower-functioning ASD to avoid undesired side-effects including behavioural activation.^94^ The knowledge of curve linear dose–response with MPH in children with lower-functioning children is crucial to avoid iatrogenic insomnia with higher doses; therefore, slow gradual titration of MPH remains key in these subgroups.^94^

When sleep disruption results from stimulant treatment that cannot be discontinued or the dosage flexibly lowered, or if stimulant rebound is determined after considering other sleep disruption causes, then adding small doses of immediate-release stimulant is a realistic option. With atomoxetine-related sleep disruption, the option is adding melatonin.^95^ Evening doses of atomoxetine and extended-release guanfacine represent other options to control daytime ADHD symptoms, with evening somnolence a common side-effect. Evening rather than morning dosing with atomoxetine has been found to reduce daytime somnolence.^37^

Given the prevalence of low ferritin levels in individuals with ASD/ADHD/sleep disruption and other neurodevelopmental disorders,^96^ ferritin should be assessed and low levels treated accordingly, especially when restless sleep or hemochromatosis is reported.^22,66,97–99^ If ferritin is below 50 ng/mL, then iron supplementation is recommended. Gabapentin has also been recommended for children and adolescents,^100^ but the evidence base is limited. However, the absence of clinical trials has not prevented gabapentin from becoming the treatment of choice for paediatric RLS as it is for adult RLS.^65^

Engaging the ECS in regulating ASD metabolic and cellular pathways with cannabidiol (CBD) is another type of intervention that has been briefly reviewed.^22^ A recent open-label CBD-rich cannabis study demonstrated significant improvements in social symptoms and repetitive and ritualistic behaviour of youth with ASD. Sleep disruption was not considered, but findings of decreases in symptoms related to worsening sleep disruption suggest the value of additional double-blind placebo-controlled studies using standardised assessments.^101^ The overall quality of evidence for CBD in the treatment of psychiatric disorders is weak,^102^ and its use is not the standard of care.

Additional considerations too numerous to cover include the dual orexin receptor agonist suvorexant that inactivates wakefulness, antipsychotics, antidepressants, anticonvulsants, antihistamines, doxepin and clonazepam.^52,64–67,99^ Hypnotics should be limited to short-acting agents for short-term use in severe SOL insomnia, and they should be used only for the shortest time and at the lowest dose.^37^ Similarly, antipsychotics should never be used as a first line of treatment of insomnia in children or adults,^103^ although sleep disruption may be a secondary benefit when irritability and aggression in ASD/ADHD/sleep disruption are controlled.

Quetiapine is often used as a hypnotic sleep agent in child psychiatry units; however, as with other antipsychotics, it is not considered a first-line choice owing to adverse metabolic effects.^64^ The alpha-agonists clonidine and guanfacine have been demonstrated as useful in managing sleep disruption in neurodevelopmental disorders.^4,20^ Difficulty with settling down at night or hyperarousal is a common problem. Clonidine and guanfacine may be useful for sleep initiation and reducing ADHD symptoms that interfere with sleep onset.^64,67^

In summary, significant advances in pharmacological interventions allow clinicians a panoply of options. However, for a precision medicine, evidence-based approach, additional randomised controlled trials are needed to support the management of sleep disorders in ASD/ADHD. This would be expected to include consideration of genetics, biomarkers and other clinical predictors of adverse effects and response to allow treatment to be tailored to individual patients’ needs.^95,104^

## Conclusions

A very high prevalence of ADHD in patients with ASD and sleep disruption and a high prevalence of ASD symptoms in patients presenting with ADHD diagnoses are documented in clinical populations. Both sets have conventionally been treated separately. Knowledge gaps with limited research describe the conundrum facing clinicians treating children, adolescents and adults with ASD/ADHD/sleep disruption symptoms. Clinicians should thoroughly assess ASD symptomatology in youth with ADHD, and *vice versa*, who present with sleep disruption to understand the special challenges for optimally guided treatment planning. Multi-treatment modalities should combine psychosocial and biological interventions to address the complexities of each case. Given the interwoven neurobiology and commonly shared genetic risk factors of the individual conditions, the assessment of ASD/ADHD/sleep disruption requires comprehensive evaluation employing subjective and objective measures as appropriate. Several intrinsic factors, e.g. the presence and absence of sensory integration disorders, should inform the choice of clinical interventions. Understanding the interactions of these risk factors, other co-occurring primary or secondary sleep disruption, medical conditions and family psychoeducation are essential tenets of treatment planning. This review updates and closes current knowledge gaps and raises unanswered questions for further empirical research. Several reviews list as limitations the need for further ASD/ADHD/sleep disruption research, including implicit bias of narrative reviews, heterogeneous populations, lack of consensus in the literature and on interventions, limited available published material, studies with highly narrow phenotypes of ASD/ADHD/sleep disruption and variability in treatment response that affect generalisability from results. A pragmatic, individualised, biopsychosocial approach must be used for optimum outcomes. To address these issues, appropriately powered future studies may yield better results. Brain endophenotypes and brain–behaviour relationships for ASD, ADHD and ASD/ADHD/sleep disruption probands and their unaffected siblings may inform clinicians, provide insight and aid intervention planning through precision psychiatry.^95,104^

## Data Availability

The materials supporting the findings are available from the relevant databases.
